# “On-The-Fly” Non-Adiabatic Dynamics Simulations on Photoinduced Ring-Closing Reaction of a Nucleoside-Based Diarylethene Photoswitch

**DOI:** 10.3390/molecules26092724

**Published:** 2021-05-06

**Authors:** Dong-Hui Xu, Laicai Li, Xiang-Yang Liu, Ganglong Cui

**Affiliations:** 1 College of Chemistry and Material Science, Sichuan Normal University, Chengdu 610068, China; 20191201049@stu.sicnu.edu.cn (D.-H.X.); lilcmail@163.com (L.L.); 2 Key Laboratory of Theoretical and Computational Photochemistry, Ministry of Education, College of Chemistry, Beijing Normal University, Beijing 100875, China

**Keywords:** photochromism, nucleoside-based diarylethene photoswitch, excited states, non-adiabatic dynamics simulation, photoinduced ring closing reaction

## Abstract

Nucleoside-based diarylethenes are emerging as an especial class of photochromic compounds that have potential applications in regulating biological systems using noninvasive light with high spatio-temporal resolution. However, relevant microscopic photochromic mechanisms at atomic level of these novel diarylethenes remain to be explored. Herein, we have employed static electronic structure calculations (MS-CASPT2//M06-2X, MS-CASPT2//SA-CASSCF) in combination with non-adiabatic dynamics simulations to explore the related photoinduced ring-closing reaction of a typical nucleoside-based diarylethene photoswitch, namely, PS-IV. Upon excitation with UV light, the open form PS-IV can be excited to a spectroscopically bright S_1_ state. After that, the molecule relaxes to the conical intersection region within 150 fs according to the barrierless relaxed scan of the C1–C6 bond, which is followed by an immediate deactivation to the ground state. The conical intersection structure is very similar to the ground state transition state structure which connects the open and closed forms of PS-IV, and therefore plays a crucial role in the photochromism of PS-IV. Besides, after analyzing the hopping structures, we conclude that the ring closing reaction cannot complete in the S_1_ state alone since all the C1–C6 distances of the hopping structures are larger than 2.00 Å. Once hopping to the ground state, the molecules either return to the original open form of PS-IV or produce the closed form of PS-IV within 100 fs, and the ring closing quantum yield is estimated to be 56%. Our present work not only elucidates the ultrafast photoinduced pericyclic reaction of the nucleoside-based diarylethene PS-IV, but can also be helpful for the future design of novel nucleoside-based diarylethenes with better performance.

## 1. Introduction

Photochromism in molecular systems refers to a photoinduced reversible transformation between two isomers having distinct absorption spectra. In addition to the color change, such a reaction is often accompanied with changes in some other physical/chemical properties such as fluorescence emission, geometry structure as well as chemical reactivity, et cetera, which gives it various applications in the fields of optical materials, memories, photoswitches and biological systems [1,2,3,4,5,6,7,8,9,10,11,12,13,14,15,16,17,18]. Motivated by their wide applications, several photochromic families such as azobenzenes, furylfulgides, spiropyrans, diarylethenes, et cetera, have been developed, in which the azobenzenes and furylfulgides change structures via *Z*/*E* isomerization while spiropyrans and diarylethenes transform between their open and closed forms via ring closing and opening reactions [9,10,11,12,13,14,15,16,17,18]. Among these photochromic families, diarylethenes, usually consisting of two aryl groups linked by a C=C double bond, are considered to be one of the most popular star molecules due to their good photoconversion quantum yield, excellent thermal stability, high fatigue resistance and fast photoresponsivity [19,20,21,22,23,24,25,26,27].

**Figure 1:**
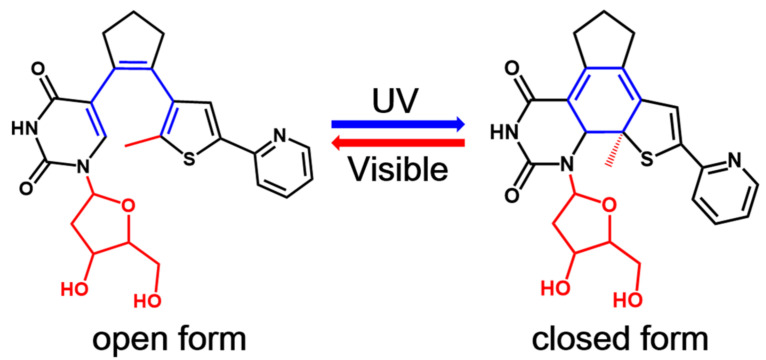
The general mechanism for the reversible photochromism process in a nucleoside-based diarylethene PS-IV. The methyl group connected to the thiophene moiety and the deoxyribosyl group of the deoxyuridine moiety (both colored in red) are replaced by hydrogen atoms in our subsequent studies to save computational efforts.

One of the most exciting potential application of these photochromic systems is their incorporation into biomolecules such as proteins and nucleic acids to modulate the corresponding biological properties with high spatio-temporal resolution using the noninvasive light [2,3,4,11,12,13,27,28,29,30,31]. Therefore, several photochromic systems including diarylethene derivatives have been successfully incorporated into biomolecules as photoswitches in the past decade [2,3,4,11,12,13,27,28,29,30,31]. In particular, Jäschke’s group designed a series of nucleoside-based diarylethene photoswitches where a nucleoside is one of the aryl group of these diarylethenes [32,33,34,35]. In 2010, they first reported a diarylethene photoswitch in which one of the two aryl groups of a typical diarylethene derivative, namely, bis(2-thienyl)ethene, is replaced by 7-deaza-8-methyldeoxyadenosine [32]. This newly synthesized photochromic nucleoside undergoes a highly efficient and reversible electrocyclic rearrangement, and the switching wavelength could be tuned by the chemical nature of substituents. However, such a photochromic molecule and its derivatives suffer from poor conversion efficiency in aqueous solvents, which severely prevents their future applications in oligonucleotides. To overcome this shortcoming, the same group later on developed another photoswitchable nucleoside in which a deoxyuridine is used as one of the aryl rings instead (see Figure 1) [34]. This novel deoxyuridine-based diarylethene photoswitch (denoted as PS-IV in previous experimental work [36,37]) and its derivatives can be easily incorporated into oligonucleotides, making them promising candidates to control DNA structures at microscopic resolution [34,35]. As depicted in Figure 1, the open form of PS-IV exhibits a pericyclic reaction upon irradiation with ultraviolet (UV) light, generating the closed form of PS-IV, which reverts back to its open form upon excitation with visible light.

An in-depth understanding of relevant photophysical and photochemical processes of photoswitches is of fundamental importance for the design of novel photoswitches with better performances. Therefore, many time-resolved spectroscopical works have been carried out to explore the underlying photochromic mechanism of various diarylethenes [38,39,40,41,42,43,44,45,46,47,48,49,50,51,52,53,54,55,56]. Particularly, Jäschke’s group applied femtosecond transient absorption spectroscopy to study the photoinduced pericyclic and cycloreversion reactions of PS-IV [36,37]. Based on the obtained results, they proposed that both reactions occur in an ultrafast manner within 500 fs and the quantum yields for the pericyclic and cycloreversion reactions of PS-IV are estimated to be 30% and 44%, respectively [36,37]. Even though these experimental works have provided valuable insights into the photochromic mechanism of PS-IV, the details of relevant reactions at microscopic level remain to be elucidated with the aid of theoretical calculations.

Despite many theoretical investigations having been performed before to explore the photochromic properties of diarylethenes, most of them are concentrated on the ground state structures as well as relevant absorption spectroscopic properties [57,58,59,60,61,62,63,64,65,66,67,68,69,70,71,72,73,74,75,76,77,78,79]. In contrast, few theoretical works concerning the microscopic details of the photochromic mechanism of diarylethenes have been conducted [80,81,82,83,84,85,86,87,88]. Such researches on the photochromic mechanisms of diarylethenes can be traced back to the beginning of the 21st century. Robb’s group first identified the importance of the conical intersections in the photochromism of diarylethenes using the CASSCF method [81], which is subsequently confirmed by more accurate CASPT2//CASSCF calculations carried out by Asano et al. [82] In 2013, Perrier et al. compared the photocyclization reactions of inverse and normal dithienylethenes using CASPT2//CASSCF methods and explained the origins for their different experimentally-observed relative efficiencies. Recently, our group also employed static electronic structure calculations (DFT/MRCI, RI-CC2, TDDFT, CASPT2//CASSCF, etc.) in combination with a semi-empirical method (OM2/MRCI) based on non-adiabatic dynamics simulations to explore the photochromic mechanism of bridged diarylethenes, based on which the experimentally proposed photochromic mechanism is refined [40,87].

Even though there have been several theoretical studies focused on the photochromic mechanism of diarylethenes, as far as we know, no computational work has been conducted to investigate the microscopic mechanism of the nucleoside-based diarylethenes. In this work, we first employed static electronic structure calculations in combination with non-adiabatic dynamics simulations to delve into the relevant structures, absorption spectra properties, photophysics and photochemistry of a typical nucleoside-based diarylethene developed by Jäschke et al., namely, PS-IV. Based on the obtained results, the relevant photochromic mechanisms of PS-IV at atomic level are clarified for the first time.

## 2. Results and Discussion

**Figure 2:**
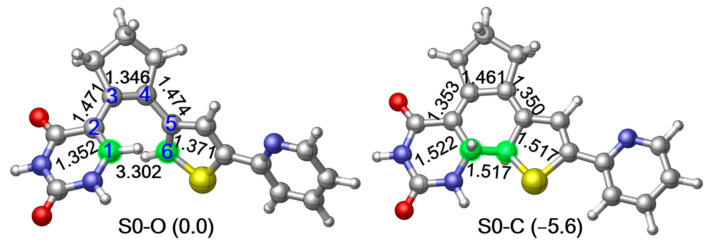
M06-2X/6-31G* optimized ground state structure and the relative energies refined at MS-CASPT2//M06-2X level of the simplified structure of PS-IV in open form (left, S0-O) and closed form (right, S0-C) respectively. Relevant bond lengths in angstrom are also shown.2. Results and Discussions.

As the first step to clarifying the photochemistry of PS-IV upon excitation, we optimized the ground state structures of the simplified PS-IV in both open and closed forms using the M06-2X/6-31G* method [89,90,91,92], which are denoted as S0-O (left panel) and S0-C (right panel), respectively, in Figure 2 (see Figure S3, Supplementary Materials for S_0_ structures optimized using the SA-CASSCF(12,10)/6-31G* method). At the MS-CASPT2//M06-2X level, the potential energy of S0-C is about 5.6 kcal/mol lower than S0-O, indicating that S0-C is slightly more stable than S0-O. In this work, we use notation, such as MS-CASPT2//M06-2X, to represent that the single point energy is refined with the MS-CASPT2/6-31G* method using the geometry optimized at the M06-2X/6-31G* level. Structurally, the most significant difference of S0-O and S0-C is the distance between the C1 and C6 atoms, which decreases from the 3.302 Å to 1.517 Å due to the ring closing reaction. Additionally, C1–C2, C3–C4 and C5–C6 increase from 1.352 Å, 1.346 Å and 1.371 Å to 1.522 Å, 1.461 Å and 1.517 Å, while C2–C3 and C4–C5 decrease from 1.472 Å and 1.483 Å to 1.354 Å and 1.328 Å, respectively. These changes in bond lengths are consistent with the bond order alterations depicted in Figure 1, where C1–C2, C3–C4 and C5–C6 change from double bonds to single bonds, and C2–C3 and C4–C5 change from single bonds to double bonds in unison with the bond ring closing reaction. Mayer bond order analysis shown in Table 1 further confirms this hypothesis, in other words, the bond order of C1–C2, C3–C4 and C5–C6 decrease from 1.663, 1.840 and 1.596 to 0.939, 1.141 and 0.964, while that of C1–C6, C2–C3 and C4–C5 increase from 0.004, 1.026 and 1.000 to 0.972, 1.710 and 1.683.

**Figure 3:**
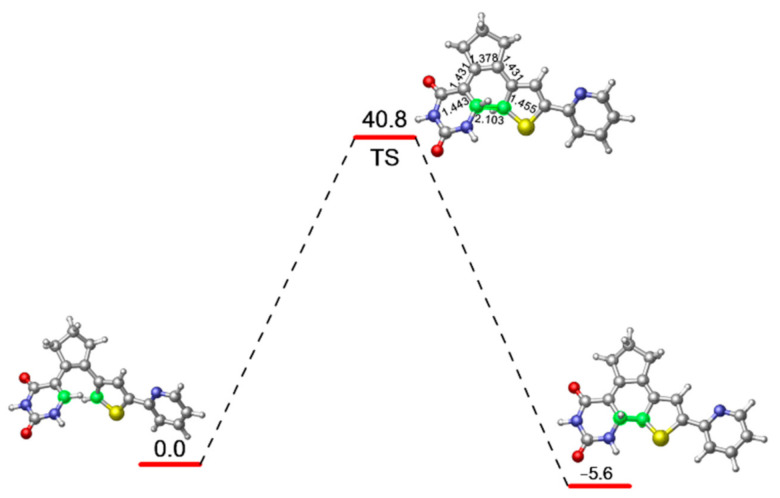
M06-2X/6-31G* optimized transition state structure connecting the open and closed forms of PS-IV as well as its relative energy refined at the MS-CASPT2//M06-2X level.

In order to evaluate the energy barrier for the ring closing reaction of PS-IV in ground state, we further optimized the related transition state structure at M06-2X/6-31G* level, as shown in Figure 3. As expected, the distance between C1 and C6 atoms becomes 1.945 Å, which is shorter than the 3.302 Å in open-form PS-IV, but longer than the 1.517 Å in closed-form PS-IV. Other relevant bond lengths also fall between the structural parameters of S0-O and S0-C. At the MS-CASPT2//M06-2X level, the energy barrier for the ring closing reaction at ground state is calculated to be 40.8 kcal/mol, which indicates that the ring closing reaction can hardly take place efficiently at room temperature, which is understandable since the diarylethenes are known for their thermal stabilities.

**Figure 4:**
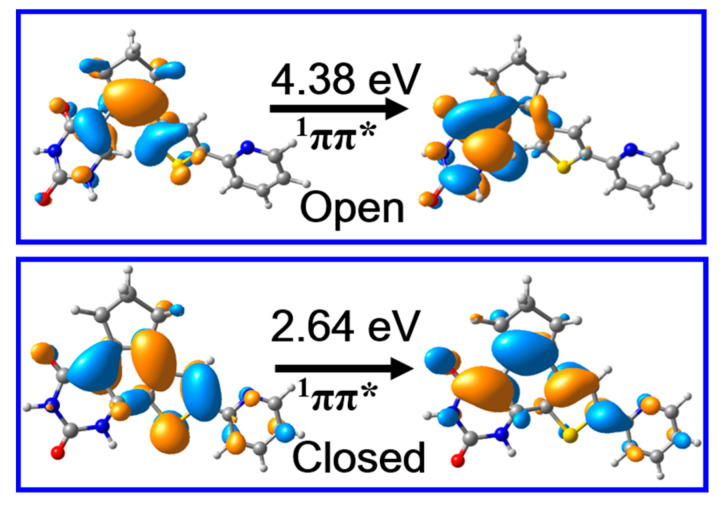
The molecular orbitals responsible for the S_0_ *→* S_1_ electronic transition and excitation energies of PS-IV in open and closed form calculated at Frank–Condon points, i.e., S0-O and S0-C, respectively, using the MS-CASPT2//M06-2X method.

Despite these ground state structures and relevant potential energies of PS-IV being highly important, the photochemistry of PS-IV are always closely related with their excited state properties. Therefore, we calculated the vertical excitation energies and corresponding oscillator strengths of the lowest lying singlet states, which can be correlated with the absorption spectra of PS-IV. Our results indicate that the S_1_ states of S0-O and S0-C are both π → π* transitions (Figure 4), and the corresponding oscillator strengths are 0.11 and 0.52, respectively, which are bright states in absorption spectra. Besides, the vertical excitation energies of open and closed forms of PS-IV are 4.38 eV (~283 nm) and 2.64 eV (470 nm), which lie in the range of UV and visible region of electromagnetic spectrum, respectively. These calculated results are in qualitative agreement with the experimentally measured first maximum absorption peak of PS-IV in methanol, namely, ~3.94 eV (315 nm) for open form and ~2.64 eV (470 nm) for closed form [36,37]. As can be seen, even though the calculated vertical excitation energies are in qualitative agreement with the experimental data, the excitation energy of the open forms are much larger than the experimental data, namely, 4.38 eV vs. 3.94 eV. There could be many reasons that result in such serious disagreement, such as the simplification of the model system, the lack of the solvent effects, the ignorance of the nuclear dynamics, the size of the basis sets, the root in the SA-CASSCF calculations, et cetera. Among these factors, the lack of solvent effects might cause the significant blue shift of the absorption spectrum. Therefore, we additionally performed calculations including the methanol solvent effects with the PCM model. The calculated vertical excitation energy turns out to be 4.14 eV and 2.55 eV, respectively, which is very close to the experimental data (within 0.2 eV). Therefore, we believe that the lack of solvent effects might be one of the most important reasons for causing such a significant discrepancy. Therefore, the influences of the environment effects on the excited state properties of PS-IV will be one of the focuses of our subsequent works, which is beyond the scope of our present work. Based on these results, we can safely postulate that the S_1_ states are the initially populated excited states upon excitation in both open and closed forms. In addition, the qualitative agreement of vertical excitation energies between calculation and experiment also validates that the methods employed here are adequate to describe the excited state properties of PS-IV.

**Figure 5:**
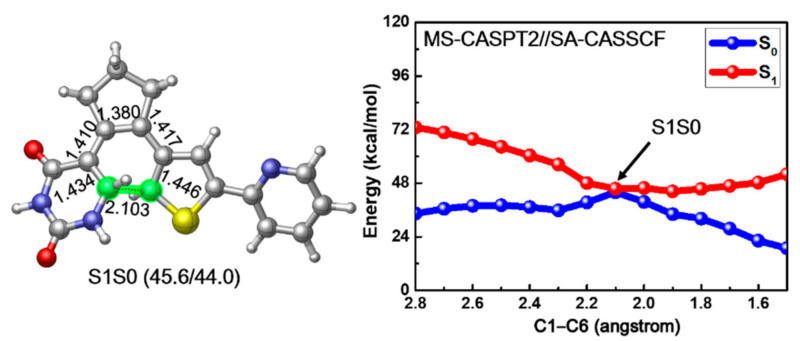
The obtained conical intersection structure S1S0 (**left**) and MS-CASPT2//SA-CASSCF calculated S_1_ relaxed scan of the C1–C6 bond connecting the PS-IV in open and closed form (**right**). Relevant bond lengths in angstrom are also shown.

Since previous vertical excitation calculations reveal that the S_1_ state is initially populated upon photoexcitation, we tried to locate the corresponding S_1_ minimum energy structure of PS-IV. However, all our optimization attempts led directly to a conical intersection structure between S_1_ and S_0_ states, which is denoted as S1S0 in the left panel of Figure 5. In comparison with the ground structures of PS-IV, we found that the bond length of C1–C6, ca. 2.103 Å, is larger than the closed form (1.517 Å) but smaller than the open form (3.302 Å). Actually, the structure of S1S0 is overall very similar to the transition state structure for the ring closing reaction of ground state, in other words, the differences between all the cyclization involved bonds in S0-TS and S1S0 are less than 0.02 Å, indicating the important role of this conical intersection in the photocyclization reaction. The energies between the S1S0 structure and S0-TS are also very close to each other, namely, 40.8 vs. 44.0 kcal/mol, implying that the deactivation from the S1S0 conical intersection might directly lead to the transition structure in ground state. This similarity has also been found in some other photochromic systems, such as B_20_H_18_^2−^ [93]. In addition, since the optimization from S0-O leads directly to such conical intersection, the relaxation from Franck–Condon region to the conical intersection region might be barrierless, which is crucial for the ultrafast non-adiabatic deactivation of the excited states. To verify this postulation, we conducted constrained optimization along the ring closing reaction coordinates, in other words, C1–C6 distance, and the corresponding relaxed scan of the C1–C6 bond is depicted in the right panel of Figure 5. Along with the decrease of the C1–C6 distance, the potential energy of the S_1_ state decreases monotonously from 73.0 to 45.6 kcal/mol, and the corresponding S_0_ potential energy increases from 34.4 to 44.0 kcal/mol, reaching the conical intersection S1S0 when the C1–C6 bond equals ca. 2.1 Å. After that, the S_1_ potential energy grows back to 52.0 kcal/mol and the S_0_ energy decreases to 18.9 kcal/mol, respectively, as the C1–C6 bond decreases to 1.5 Å, corresponding to the closed form of PS-IV. These results not only prove that the relaxation from the Franck–Condon region to the S1S0 conical intersection is barrierless, but also suggest that neither the ring closing reaction from the open form nor the ring opening reaction from the closed form of PS-IV can complete in the S_1_ state alone due to the up-hill tendency to reach the closed (for ring closing reaction) and open (for ring opening reaction) forms of PS-IV in the S_1_ surface from the S1S0 conical intersection. Instead, the reactions are more likely to complete in ground state following the ultrafast internal conversion from S_1_ to S_0_. Finally, a Mulliken charge analysis along the reaction path indicates that the ring closing reaction in the S_1_ state is accompanied with a charge transfer process, as shown in Figure S5, Supplementary Materials. The electron gradually transfers from the thiophene-pyridine fragment (FRAG-2, in red) to the uracil-cyclopentene fragment (FRAG-1, in blue) during the photocyclization.

These static electronic structure calculations have provided valuable insights into the photoinduced ring-closing reaction mechanism of PS-IV, however, dynamical properties including relevant timescales, structural changes and branching ratios remain unclear. In order to clarify these issues, we have conducted Zhu–Nakamura method based non-adiabatic dynamics simulations. Since the potential energy profile along the ring-closing reaction coordinate calculated at the SA-CASSCF level is overall similar to that obtained using MS-CASPT2//SA-CASSCF calculations (see Figure S4, Supplementary Materials and Figure 5), we employed the SA-CASSCF method to calculate the energies and gradients needed in the non-adiabatic dynamics simulations to save computational efforts. In order to simulate the ring-closing reaction, the initial conditions (geometries and velocities) are obtained via Wigner sampling of the open form PS-IV [94,95,96,97,98,99,100]. Additionally, only two states, namely, S_0_ and S_1_, are included in our simulations, from which the S_1_ state is selected as the initial state. Finally, a total of 40 trajectories are propagated for 500 fs with a time step of 1.0 fs and 34 successfully ended trajectories are carefully analyzed to obtain final results. All these trajectories decay to ground state at the end of the 500-fs simulation time.

**Figure 6:**
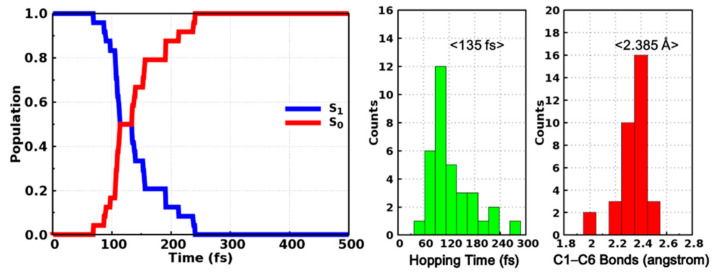
Time-dependent state population of the S_0_ and S_1_ states (**left**) and the distributions of hopping time and hopping structural parameters (**right**).

The averaged state populations of the S_0_ and S_1_ states are shown in the left panel of Figure 6. Even though the curves are not smooth enough considering our limited trajectories, it is obvious that the population of the S_1_ state decreases monotonously while that of the S_0_ state increases monotonously at the same time. The population of S_1_ (S_0_) decreases (increases) from 1.0 (0.0) to almost 0.0 (1.0) within 200 fs in our simulation, which implies that the internal conversion from the excited S_1_ state to the S_0_ state is accomplished in an ultrafast manner. The time constant is estimated to be 143 fs through fitting the S_1_ population with a single exponential decay function *y* = *exp*(*−x/t*). Moreover, we have also counted the distributions of hopping time as well as the C1–C6 bond length of the hopping structures, as depicted in the right panel of Figure 6. Most trajectories hop to ground state within 180 fs and the maximum appears at around 120 fs. The averaged hopping time is 135 fs, which is consistent with the time dependent populations of the S_0_ and S_1_ states. The C1–C6 bond lengths of hopping structures are mostly located in the range from 2.0 to 2.5 Å (Figure 6) and the energy difference between the S_1_ and S_0_ states of these structures are distributed in the range from 0 to 4 kcal/mol (Figure S6), which are exactly around the conical intersection, indicating that even though these hopping events are closely related with the ring closing reactions, the photocyclizations are completed after hopping to the ground state rather than in the S_1_ state alone. These dynamical results are consistent with our previous static electronic structure calculations and the ultrafast internal conversion can be attributed to the barrierless pathway to the conical intersection region as indicated by the relaxed scan of the C1–C6 bond in the S_1_ state (Figure 5).

**Figure 7:**
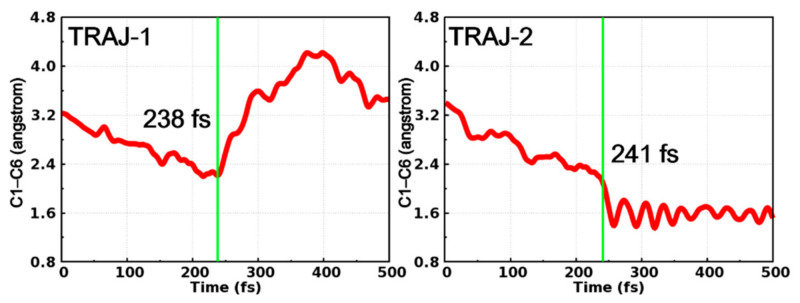
Time-dependent C1–C6 distance in two typical trajectories starting from the open form. Their hopping times (green lines) are also shown.

After in-depth analysis of all successfully ended trajectories, we found that these trajectories can be divided into two categories according to the time dependent evolution of C1–C6 distance, as depicted in Figure 7. At the early stage of the simulations, the dynamical behaviors of these two trajectories are quite similar to each other, in other words, both trajectories witness the decrease of the C1–C6 distance before hopping to the ground state at 238 fs (TRAJ-1) and 241 fs (TRAJ-2), respectively. Furthermore, the behaviors of the two trajectories become different. For TRAJ-1, the C1–C6 distance increases to more than 3.0 Å, indicating that the trajectory returns to the open form of PS-IV. However, for TRAJ-2, the C1–C6 distance continues to decrease to ca. 1.6 Å and fluctuates there, which corresponds to the formation of the closed form of PS-IV.

**Figure 8:**
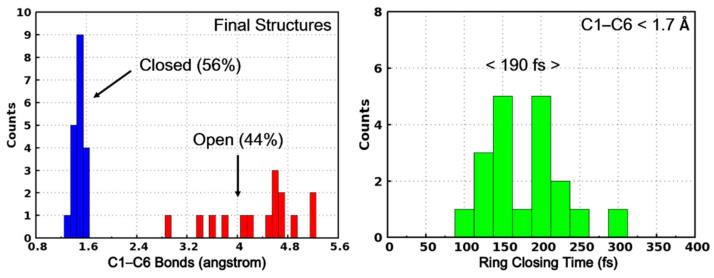
The C1–C6 bond distribution of the final structures of the surface hopping trajectories. The structures with C1–C6 less than 1.70 Å are regarded as PS-IV in closed form (in blue) while others are PS-IV in open form (in red).

Based on the two kinds of trajectories, we can also categorize the final structures into two classes according to their C1–C6 distance. As shown in Figure 8, the final structures with C1–C6 distance shorter than 1.70 Å are regarded as photocyclization products (PS-IV in closed form) while the others are the initial open-form reactants. Based on this classification, the quantum yield of the ring-closing reaction upon photoexcitation is calculated to be 56%. This predicted quantum yield is slightly higher than the previously experimentally estimated value of 44% [36,37], which can be attributed to the limited trajectories, the simplified model system and the lack of considering environmental effects in our present work. Furthermore, we have also counted the distribution of the ring-closing times of those trajectories leading to the products, as shown in the right panel of Figure 8, where the time of the first occurrence of C1–C6 distance less than 1.70 Å is regarded as the ring-closing completing time. As can be seen, most of the final products are formed within 300 fs and the averaged time is 190 fs. Therefore, after the internal conversion from S_1_ to S_0_ (~143 fs), the ring-closing reaction completes in about 60 fs.

**Figure 9:**
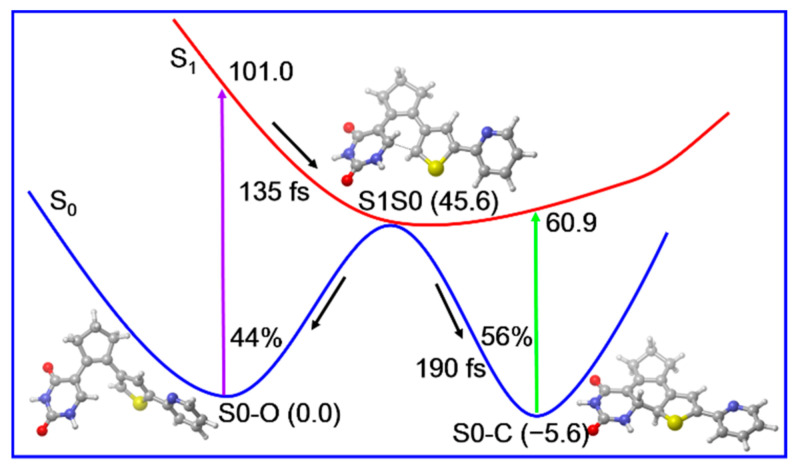
Proposed photoinduced ultrafast ring-closing reaction mechanism of PS-IV based on our static electronic structure and non-adiabatic dynamics simulations. The relative energies (in kcal/mol) and timescales (in femtosecond) are also shown.

According to those static electronic structures and non-adiabatic simulation results, we suggested the photocyclization reaction mechanism of the open form PS-IV in Figure 9. The open form PS-IV can be excited to the spectroscopically bright S_1_ state upon irradiation with UV light. After that, the photoexcited molecules relax from the Franck–Condon region to the conical intersection region between S_1_ and S_0_ with a time constant of about 143 fs due to the barrierless relaxed scan of the C1–C6 bond, from which the molecules decay to the ground state immediately. Once reaching the ground state, the molecules can either return to the open-form reactant or generate the closed-form products in an ultrafast manner (~190 fs). Finally, the quantum yield for the photocyclization reaction is estimated to be 56% and all other trajectories return to the open form reactant.

Finally, we need to emphasize that as the first theoretical work concerning the photoinduced processes of these novel nucleoside-based diarylethene photoswitches, we have used the most simplified model system to capture the most essential characteristics of such systems. There are many other issues remaining to be settled, such as the steric effects of the methyl group, the substitution effects and the environmental effects to the excited-state dynamics of these systems, which are the focuses of our subsequent works.

## 3. Materials and Methods

### 3.1. Electronic Structure Calculations

In order to save computational efforts, we use a simplified model of PS-IV in all our calculations, in which the methyl group connected to the thiophene moiety and the deoxyribosyl group of the deoxyuridine moiety (colored red in Figure 1) are replaced by hydrogen atoms (see Figure 2). All ground state structures, including PS-IV in open- and closed forms as well as corresponding transition state structure, are first optimized using the density functional theory (DFT) method with M06-2X functional [89,90]. All the excited state related structures and relaxed scans of the C1–C6 bond are then optimized using the SA-CASSCF method.

In order to obtain more accurate potential energy profiles, the MS-CASPT2 method is employed to refine the single-point energies of all optimized structures [101,102]. In MS-CASPT2 and SA-CASSCF calculations, 12 π-electrons are placed into the active space that is comprised of 10 π and π* orbitals (see Figures S1 and S2, Supplementary Materials) and two roots with equal weights are included in relevant SA-CASSCF calculations. In MS-CASPT2 computations, the Cholesky decomposition technique with unbiased auxiliary basis sets is used for accurate two-electron integral evaluation [103]; the ionization potential-electron affinity (IPEA) shift is not applied [104], whereas the imaginary shift technique of 0.2 a.u. is employed to avoid intruder-state issues [105]. Finally, the 6-31G* basis set is used in all kinds of involved calculations [91,92]. The 6-31G* basis set was used due to the large computational efforts in MS-CASPT2 calculations and non-adiabatic dynamics simulations considering our limited computational sources. However, this basis set is robust for small organic molecules and could provide at least qualitatively correct results for these systems as shown in our previous works [106,107,108]. As the first theoretical work investigating the photoinduced processes of nucleoside-based diarylethene, we performed all our calculations in vacuum without considering any environmental effects.

Additionally, all DFT calculations were performed using the GAUSSIAN09 package [109], while all SA-CASSCF and MS-CASPT2 calculations were conducted using the MOLCAS8.0 package [94,110]. The Mayer bond order analysis was performed using Multiwfn3.6 [111].

### 3.2. Non-Adiabatic Dynamics Simulations

Trajectory-based semi-classical non-adiabatic dynamics simulations approaches with different schemes were extensively employed to simulate a series of ultrafast excited-state relaxation processes in chemical and biological systems, and materials [87,95,96,112,113,114,115,116,117,118,119,120,121,122,123,124,125,126]. In the present work, trajectory-based surface hopping dynamics simulations involving the lowest two singlet states were carried out with the recently developed method proposed by Zhu et al. [127]. In this dynamics simulation approach, the system is merely propagated in an electronic state at any time; however, near quasi-degenerate regions, it can jump between different potential energy surfaces. The non-adiabatic transition probability is computed according to the Landau–Zener formula improved by Zhu and Nakamura (Equation (1)) [128,129,130],

(1) $$p=\exp\left(-\frac{\pi }{4\sqrt{a^{2}}}\sqrt{\frac{2}{b^{2}+\sqrt{\left|b^{4}\pm 1\right|}}}\right)$$

in which two unitless parameters, namely, effective coupling and collision energy, are written as (Equations (2) and (3)):

(2) $$a^{2}=\frac{\hbar^{2}}{2\mu }\frac{\sqrt{\left|F_{2}F_{1}\right|}\left|F_{2}-F_{1}\right|}{{\left(2V_{12}\right)}^{3}}$$

and

(3) $$b^{2}=\left(E_{t}-E_{x}\right)\frac{\left|F_{2}-F_{1}\right|}{\sqrt{\left|F_{2}F_{1}\right|}\left(2V_{12}\right)}$$

where *F*_1_ and *F*_2_ are two mass-scaled one-dimensional diabatic forces, *V*_12_ is diabatic coupling, *µ* is the reduced mass, *E_x_* is the energy at the crossing point, and *E_t_* is the potential energy plus kinetic energy component along the hopping vector direction. Finally, the mass-scaled one-dimensional diabatic forces in Equations (2) and (3) are converted from mass-scaled multi-dimensional diabatic forces based on Equations (4) and (5):

(4) $$\frac{\sqrt{\left|F_{2}F_{1}\right|}}{\sqrt{\mu }}=\sqrt{\left|\overset{N}{\underset{i=1}{\sum }}\frac{1}{m_{i}}\underset{\alpha =x.y.z}{\sum }F_{2}^{i\alpha }F_{1}^{i\alpha }\right|}$$

and

(5) $$\frac{\left|F_{2}-F_{1}\right|}{\sqrt{\mu }}=\sqrt{\left|\overset{N}{\underset{i=1}{\sum }}\frac{1}{m_{i}}\underset{\alpha =x,y,z}{\sum }{\left(F_{2}^{i\alpha }-F_{1}^{i\alpha }\right)}^{2}\right|}$$

in which *N* is the number of nuclei in a system, m*_i_* is the *i*-th atomic mass, $F_{1}^{i\alpha }$ and $F_{2}^{i\alpha }$ are multidimensional diabatic forces of the *i*-th atom (α: *x*, *y* and *z*) related to the involved two states. These diabatic forces can be further converted from multi-dimensional adiabatic forces, which are directly calculated by the several electronic structure packages. The detailed description of the definitions of the hopping direction and the momentum change at a hopping region can be found in recent works by Zhu and co-workers [97,127]. This Zhu–Nakamura non-adiabatic dynamics method has recently been coded and implemented as a module into our own generalized trajectory-based surface hopping (GTSH) package [98,99]. Compared with the fewest-switches surface-hopping method developed by Tully and Preston [96,124], the largest advantage of the Zhu–Nakamura method is that it does not need to compute expensive non-adiabatic coupling vectors, in particular for those electronic structure methods incapable of efficiently computing these vectors.

Initial atomic coordinates and velocities in our non-adiabatic dynamics simulations are obtained via the Wigner sampling method [100], as done in previous works by us and others [112,120,126,131,132,133]. A total of 40 surface-hopping trajectories are run starting from the initially populated bright excited singlet state, in other words, ^1^ππ* with all relevant energies and gradients being computed on-the-fly as needed. For points with energy gaps less than 30 kcal/mol, improved Landau–Zener formula in Equation (1) is applied to compute non-adiabatic transition probability, which is used to decide whether to hop or not. A time step of 1.0 fs was chosen for nuclear propagation by numerically integrating the Newtonian equation of motion using the velocity Verlet algorithm [134] and a total of 500 fs was propagated for each trajectory. Final evaluations were done for 34 trajectories that successfully finished in our non-adiabatic dynamics simulations. All quantities needed in our non-adiabatic dynamics simulations, namely, energies and gradients, were computed at SA-CASSCF level through the external MOLCAS8.0 package [94,110].

## 4. Conclusions

In summary, in this work we first employed static electronic structure calculation and non-adiabatic dynamics simulations to investigate the photoinduced properties of a nucleoside-based diarylethene photoswitch PS-IV. Our calculation results indicate that upon excitation to the S_1_ state, PS-IV can relax in an ultrafast manner to the conical intersection region via a barrierless pathway, from which the internal conversion takes place immediately. The timescale of the internal conversion to ground state is estimated to be ca. 143 fs using these trajectories. Even though the conical intersection, which holds a structure similar to the ground state transition structure connecting the open and closed form of PS-IV, plays an important role in promoting the photoinduced ring closing reaction of PS-IV, the analyses of the relaxed scan of the C1–C6 bond and hopping structures indicate that the ring closing reaction cannot complete in the S_1_ state alone. After relaxing to ground state, the molecules either return back to the initial open form of PS-IV (44%) or exhibit pericyclic reaction to generate the closed form of PS-IV within 100 fs (56%). Our simulation results not only elucidate the relevant photoreaction mechanisms of PS-IV, but can also be helpful for the future design of novel nucleoside-based diarylethene photochromic systems.

## Figures and Tables

**Table 1:** Mayer bond order analysis of ground structures of PS-IV in open form (S0-O) and closed form (S0-C) calculated at M06-2X/6-31G* level.

Bond Orders	C1–C2	C2–C3	C3–C4	C4–C5	C5–C6	C1–C6
S0-O	1.663	1.026	1.840	1.000	1.596	0.004
S0-C	0.939	1.710	1.141	1.683	0.964	0.972

## Data Availability

The data presented in this study are available on reasonable request from the corresponding author.

## Supplementary Materials

Figure S1: Active Space of Open Form of PS-IV, Figure S2: Active Space of Closed Form of PS-IV, Figure S3: SA-CASSCF Optimized S0 Structures, Figure S4: SA-CASSCF Calculated S1 Minimum Energy Path, Figure S5: MS-CASPT2 Calculated S1 Mulliken Charges, Figure S6: Energy Difference Distributions of the Hopping Structures, Table S1: Cartesian Coordinates.

## Sample Availability

Samples of the compounds are not available from the authors.
