# Supplementary material for: “On-The-Fly” Non-Adiabatic Dynamics Simulations on Photoinduced Ring-Closing Reaction of a Nucleoside-Based Diarylethene Photoswitch
Source: Molecules. 2021 May 6;26(9):2724. doi: 10.3390/molecules26092724 (PMC8125013; doi:10.3390/molecules26092724)
Supplement: Supplementary file 1 [file molecules-26-02724-s001.zip › molecules-1199145-supplementary.pdf]

molecules

Supplementary Materials: "On-The-Fly" Non-adiabatic Dynamics Simulations on Photoinduced Ring-Closing Reaction of a Nucleoside-Based Diarylethene Photoswitch

Dong-Hui Xu [1], Laicai Li [1], Xiang-Yang Liu [1,*] and Ganglong Cui [2,*]

[1] College of Chemistry and Material Science, Sichuan Normal University, Chengdu 610068, China
[2] Key Laboratory of Theoretical and Computational Photochemistry, Ministry of Education, College of Chemistry, Beijing Normal University, Beijing 100875, China
* Correspondence: xiangyangliu@sicnu.edu.cn; ganglong.cui@bnu.edu.cn

Table of Contents

Additional Figures 2
Active Space of Open Form of PS-IV 2
Active Space of Closed Form of PS-IV 2
SA-CASSCF Optimized S_0 Structures 2
SA-CASSCF Calculated S_1 Minimum Energy Path 3
MS-CASPT2 Calculated S_1 Mulliken Charges 3
Energy Difference Distributions of the Hopping Structures 4
Coordinates 4

Additional Figures

Active Space of Open Form of PS-IV

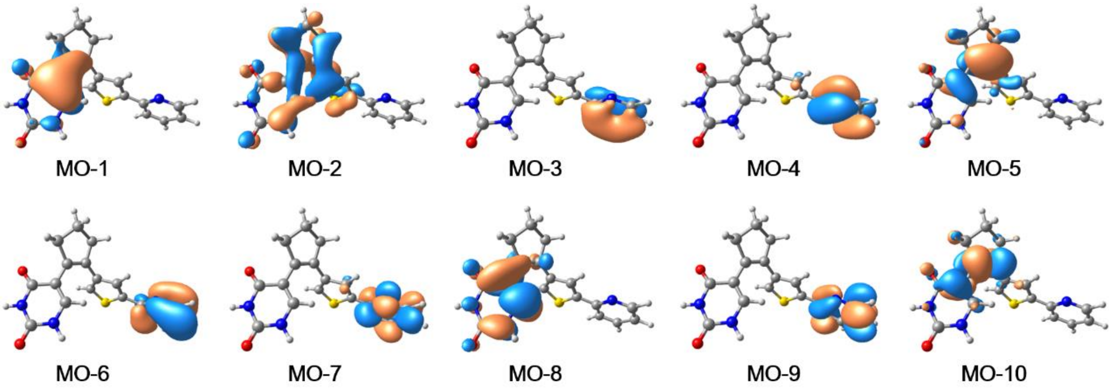

Figure S1. Molecular orbitals included in the (12,10) active space of the open form of PS-IV.

Active Space of Closed Form of PS-IV

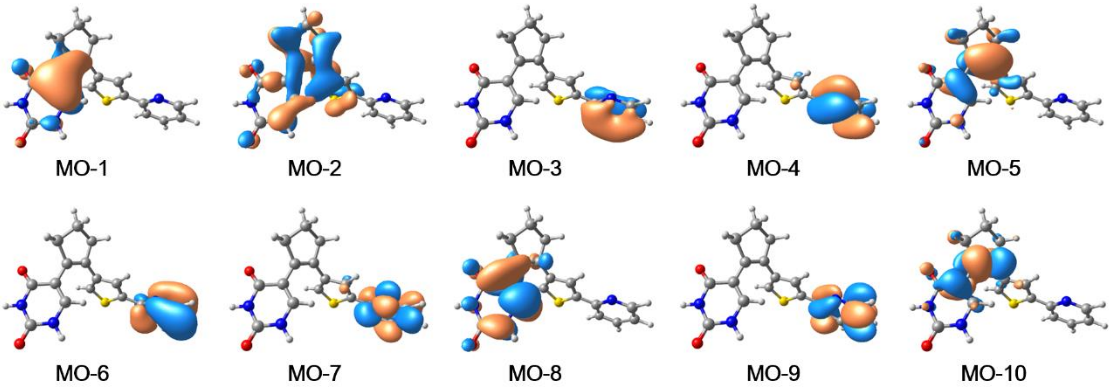

Figure S2. Molecular orbitals included in the (12,10) active space of the closed form of PS-IV.

SA-CASSCF Optimized S_0 Structures

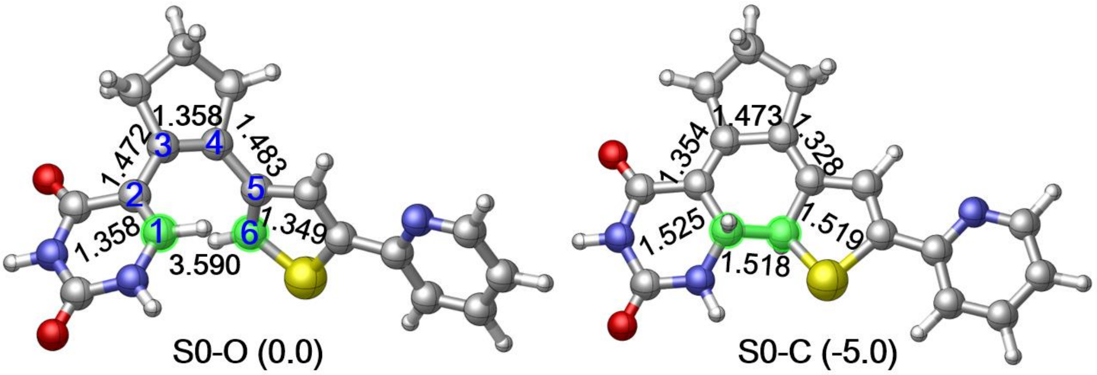

Figure S3. SA-CASSCF/6-31G* optimized ground state structures and the relative energies calculated at MS-CASPT2//SA-CASSCF level of the simplified structure of PS-IV in open form (left, S0-O) and closed form (right, S0-C) respectively. Also shown are relevant bond lengths in angstrom.

SA-CASSCF Calculated S_1 Minimum Energy Path

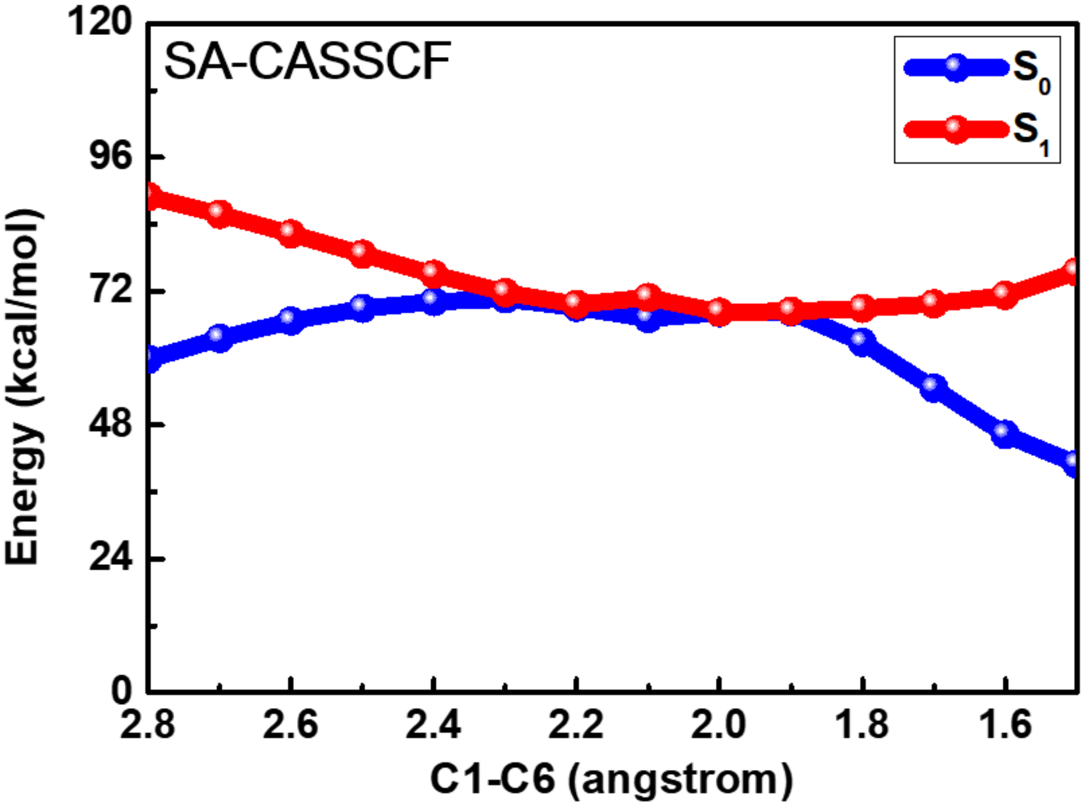

Figure S4. SA-CASSCF/6-31G* calculated S_1 relaxed scan of the C1-C6 bond connecting the PS-IV in open and closed form.

MS-CASPT2 Calculated S_1 Mulliken Charges

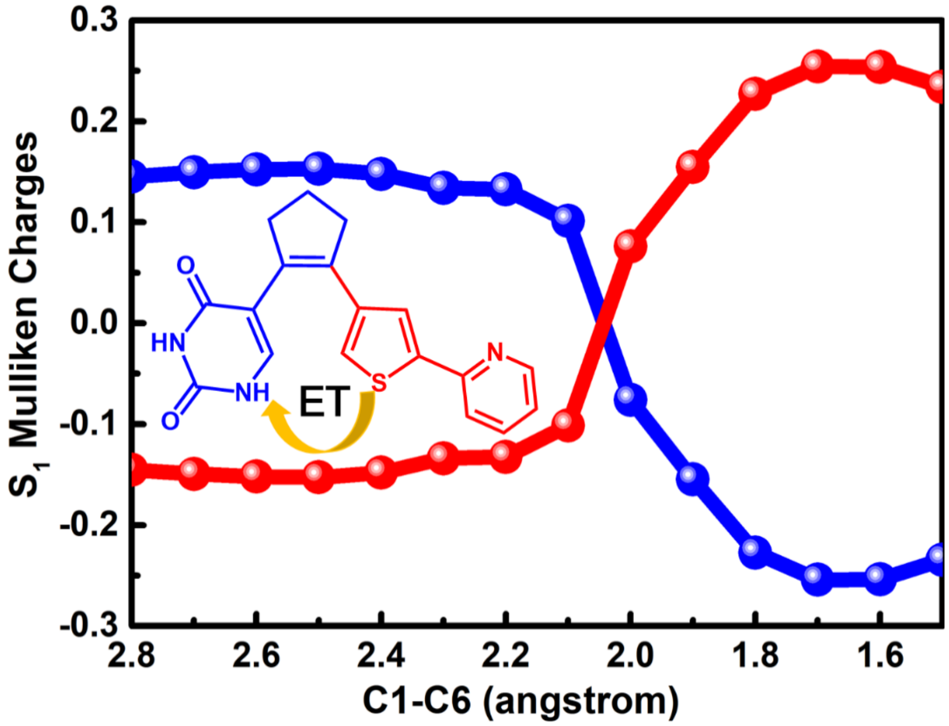

Figure S5. The S_1 Mulliken charges of uracil-cyclopentene fragment (FRAG-1, in blue) and thiophene-pyridine fragment (FRAG-2, in red) calculated at MS-CASPT2//SA-CASSCF level along the ring closing reaction coordinate.

Energy Difference Distributions of the Hopping Structures

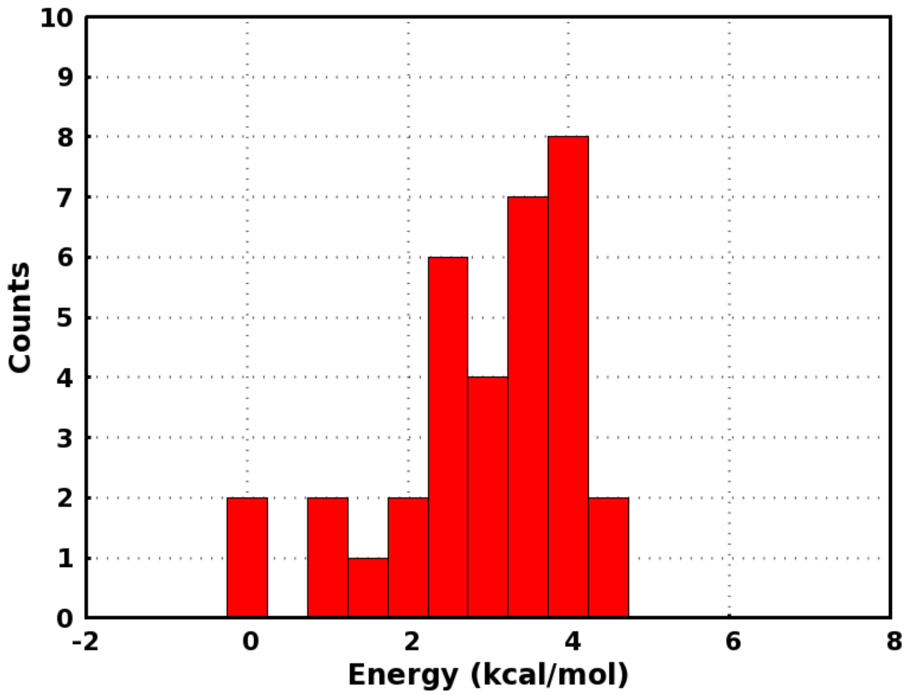

Figure S6. The distributions of energy differences between S_1 and S_0 states of the hopping structures.

Coordinates

Table S1. Cartesian Coordinates of All Optimized Structures (in xyz format).

Unit: angstrom			
39			
S0_O_DFT			
C	-5.60368602	0.53151149	-1.25790297
C	-6.47130329	-0.45540761	-0.79501788
C	-5.97569387	-1.38170825	0.11578777
C	-4.65177354	-1.28435745	0.52139466
C	-3.85882417	-0.25611107	-0.00104705
N	-4.33284043	0.63871766	-0.88102897
H	-6.61122897	-2.17020871	0.50722901
H	-5.95041491	1.27654962	-1.97100450
H	-7.49870266	-0.49137031	-1.13968475
H	-4.23888857	-1.99337864	1.23278259
C	-0.17486832	-0.25611342	1.34976748
C	-0.27811843	0.79104319	0.47031119
C	-1.59768909	0.88620723	-0.07490533
C	-2.44833328	-0.08350216	0.37866899
S	-1.64571937	-1.13025777	1.50675643
H	-1.92065781	1.63536806	-0.78810132
C	1.84779978	3.79806611	-0.47778046
C	0.57057531	3.22701606	0.16379866
C	0.81104817	1.73061501	0.14881546
C	2.10332122	1.45174182	-0.10416283
C	2.92191550	2.72185101	-0.23861680

H	2.12644558	4.77467421	-0.07483700
H	-0.34086911	3.51231032	-0.37327407
H	0.44106604	3.56600946	1.20102365
H	3.65848472	2.66103871	-1.04644448
H	3.48520346	2.89323526	0.68549347
H	1.68650485	3.91574904	-1.55439332
C	3.89779766	-2.47569368	-0.61869620
N	4.50823707	-1.44749656	0.07226857
C	4.02978928	-0.15056245	0.31206925
C	2.70639240	0.11972550	-0.26662389
C	2.10242863	-0.87002174	-0.96239025
N	2.65968785	-2.11311041	-1.12556540
O	4.69831150	0.63299326	0.95495062
O	4.38127387	-3.57496187	-0.77270200
H	5.41796581	-1.66409244	0.46453397
H	0.69523082	-0.54642503	1.92329777
H	1.12962612	-0.72890661	-1.42128761
H	2.18480246	-2.83325197	-1.65196356
39			
S0_C_DFT			
C	-5.70976341	0.98257800	-0.11941845
C	-6.43931960	-0.20617250	-0.17404185
C	-5.73984994	-1.40472635	-0.12980745
C	-4.35322353	-1.37030219	-0.03305815
C	-3.71376840	-0.12819070	0.01392809
N	-4.38622579	1.03298510	-0.02869896
H	-6.26213481	-2.35560583	-0.16900851
H	-6.22059837	1.94259145	-0.15136578
H	-7.52084026	-0.18278327	-0.24907657
H	-3.77884950	-2.29030104	0.00948359
C	0.25533783	-0.45452217	0.49742050
C	-0.13517480	0.98559815	0.22427886
C	-1.56239854	1.15167291	0.17223866
C	-2.24945099	-0.01224975	0.11229759
S	-1.23081427	-1.46279460	0.12078072
H	-2.06633924	2.11025004	0.13331013
C	2.20526696	3.80174071	-0.41946258
C	0.77553401	3.40216905	-0.00289507
C	0.85180610	1.89825146	0.10003584
C	2.24929685	1.47232729	0.11138721
C	3.11930625	2.70467497	0.16284646
H	2.48101919	4.80254690	-0.07988776
H	0.01290937	3.74445331	-0.70889320
H	0.52452568	3.82778088	0.97780503
H	4.06150694	2.57702201	-0.36837617
H	3.36866447	2.91913228	1.21081078
H	2.28219818	3.78795791	-1.51174051
C	3.20387732	-2.64578157	-0.08802956
N	4.16343934	-1.64265852	-0.23135112
C	3.99613021	-0.27303954	-0.05268313
C	2.59055802	0.16775228	-0.00335938

C	1.49614736	-0.85280442	-0.27865592
N	1.92473832	-2.18728743	0.10888107
O	4.96588621	0.45524834	0.04178772
O	3.50516894	-3.81960684	-0.10374708
H	5.12111780	-1.97318422	-0.25687239
H	0.47049655	-0.57086656	1.56994425
H	1.25066146	-0.82121478	-1.35730429
H	1.24471650	-2.93332516	0.01440786
39			
S0_TS			
C	5.53624672	0.99068258	0.58519445
C	6.32158640	-0.15052087	0.41751628
C	5.70261052	-1.31673989	-0.01476322
C	4.33514506	-1.29751930	-0.26097985
C	3.63563070	-0.10392788	-0.06172433
N	4.22826514	1.02415311	0.35598567
H	6.27241271	-2.22886084	-0.16104822
H	5.98357998	1.92280617	0.92233470
H	7.38596589	-0.11745163	0.62202426
H	3.82171873	-2.19077595	-0.60355016
C	-0.25220729	-0.40407138	-0.74050612
C	0.06114468	0.95687284	-0.37710070
C	1.43879566	1.14655411	-0.13743016
C	2.18728949	0.00132627	-0.31120154
S	1.23718650	-1.35062174	-0.80785265
H	1.88373780	2.07388927	0.20193093
C	-2.31372875	3.86611459	-0.14868933
C	-0.84262750	3.41093437	-0.00767969
C	-0.97387134	1.90890992	-0.11493259
C	-2.25545784	1.49261460	0.17136731
C	-3.15629592	2.68044826	0.36115934
H	-2.53344072	4.03551443	-1.20729859
H	-0.42624710	3.71557711	0.96380646
H	-0.19832234	3.84230659	-0.78138933
H	-3.39546915	2.76720275	1.43069651
H	-4.10762153	2.53146690	-0.15711353
H	-2.52216346	4.79544875	0.38654631
C	-2.99756557	-2.71613466	0.31103738
N	-3.94160931	-1.82317636	-0.10207158
C	-3.87139178	-0.39582347	-0.05826805
C	-2.61404298	0.11996922	0.35728562
C	-1.51672712	-0.77504059	0.69045424
N	-1.81051769	-2.11705454	0.75226375
O	-4.87190678	0.22638679	-0.39709157
O	-3.13489984	-3.92096735	0.34304292
H	-4.83988052	-2.20866150	-0.36552355
H	-0.95700861	-0.61741207	-1.53850402
H	-0.83800809	-0.45874113	1.48664717
H	-1.14995039	-2.77106421	1.15079338
39			
S0_O_CAS			

C	-11.00261129	-0.46156613	-1.65514542
C	-9.86312310	-0.67477202	-2.41881503
C	-9.12227315	0.43729940	-2.80582440
C	-9.54394755	1.68697926	-2.42017210
C	-10.70774261	1.80878021	-1.65207412
N	-11.41901498	0.74566836	-1.28133805
H	-8.23064573	0.32455488	-3.39600204
H	-11.60780547	-1.28904041	-1.33030201
H	-9.56925944	-1.66960182	-2.69684077
H	-8.98203967	2.55754693	-2.70262947
C	-11.66557862	5.52105606	-0.75944987
C	-12.53757823	4.72106710	-0.11259094
C	-12.26475007	3.32966056	-0.36838715
C	-11.22131820	3.12049268	-1.19784457
S	-10.52042871	4.63375309	-1.69311573
H	-12.83330785	2.52341360	0.05170086
C	-14.97738939	5.29764663	2.75613727
C	-13.57332335	4.90017186	2.27320920
C	-13.62126598	5.19158567	0.78422215
C	-14.68590879	5.97741825	0.47743160
C	-15.46909609	6.33527444	1.73294827
H	-14.98334846	5.67607597	3.77196602
H	-13.33645874	3.86236534	2.48694999
H	-12.79646711	5.50019725	2.74370988
H	-16.54263375	6.29235275	1.58322597
H	-15.23721756	7.34721253	2.04328513
H	-15.62531763	4.42667300	2.72923813
C	-15.92919067	7.27547902	-3.47986437
N	-16.00202943	8.08006667	-2.36718043
C	-15.63184471	7.78647823	-1.06899737
C	-15.10455112	6.41366527	-0.86495877
C	-15.04872501	5.61025150	-1.95783702
N	-15.43638414	6.03309189	-3.21660420
O	-15.73654159	8.61012903	-0.21358663
O	-16.26775323	7.63905703	-4.56585587
H	-16.35132753	9.00197182	-2.52598232
H	-11.62787953	6.59127034	-0.73839336
H	-14.68892261	4.60391208	-1.90990462
H	-15.36800890	5.42092869	-3.99797384
39			
S0_C_CAS			
C	-9.83687121	-0.10127577	-0.85144242
C	-9.33460658	-0.45866351	-2.09957885
C	-9.65384183	0.34704199	-3.18543469
C	-10.44615254	1.45434775	-2.98505920
C	-10.90909111	1.73841978	-1.69619180
N	-10.60194741	0.96385222	-0.64958924
H	-9.28864978	0.11315818	-4.16907558
H	-9.61519823	-0.69464520	0.01757691
H	-8.71947510	-1.33195173	-2.21002520
H	-10.70013639	2.09254911	-3.80974184

C	-12.99316335	5.18403033	-1.49518506
C	-13.07553362	4.42615929	-0.18169034
C	-12.23496514	3.25231955	-0.18459819
C	-11.76125349	2.90171723	-1.40525289
S	-12.31734329	3.99034130	-2.70945364
H	-12.02141748	2.67559932	0.69243128
C	-15.24546246	5.23124221	2.70980424
C	-14.01366007	4.44819423	2.21954374
C	-13.84669928	4.90670764	0.78628383
C	-14.67826920	6.10075630	0.55728783
C	-15.25295897	6.53425997	1.88897995
H	-15.22604481	5.41321409	3.77764916
H	-14.13541786	3.37511702	2.30810301
H	-13.13320550	4.72244592	2.79551920
H	-16.22806968	6.98412073	1.80390628
H	-14.59289571	7.27329734	2.33752135
H	-16.14659289	4.66551050	2.49244331
C	-15.07170277	7.62668949	-3.35759648
N	-15.86004899	8.06954199	-2.31098803
C	-15.76134887	7.73601566	-0.97879312
C	-14.88204617	6.57645638	-0.69342930
C	-14.33357241	5.78613052	-1.87696716
N	-14.18635672	6.64726730	-3.03632777
O	-16.36280687	8.36054940	-0.15644006
O	-15.16351849	8.10916003	-4.44727339
H	-16.44231126	8.85153742	-2.52477087
H	-12.27293935	5.98963864	-1.39217977
H	-15.02851061	4.97482709	-2.09666848
H	-13.75892251	6.25288728	-3.84694073
39			
S1S0			
C	-10.38755849	-0.36423504	-1.10441343
C	-9.46747492	-0.52350431	-2.13284768
C	-9.23368791	0.55661246	-2.97435168
C	-9.92163419	1.72967155	-2.76320836
C	-10.82346280	1.80023141	-1.69761010
N	-11.04829839	0.76500302	-0.88799753
H	-8.52420866	0.48018040	-3.77691763
H	-10.60580658	-1.17257135	-0.43063302
H	-8.95648386	-1.45894277	-2.26168667
H	-9.75644118	2.57611262	-3.40168840
C	-12.70459780	5.29186639	-1.45219852
C	-12.94128503	4.52737080	-0.24726827
C	-12.27686842	3.26205730	-0.27061931
C	-11.57087197	3.03485089	-1.39173799
S	-11.64329221	4.36945991	-2.53088155
H	-12.35707283	2.53361850	0.51664462
C	-15.11118104	5.24827049	2.74899791
C	-13.89451799	4.48297295	2.18808078
C	-13.83999872	4.97388839	0.75284739
C	-14.72130795	6.01472696	0.54247424

C	-15.31246641	6.48001454	1.84893130
H	-14.97593476	5.51230587	3.79050998
H	-14.00640534	3.40552076	2.26494531
H	-12.97392194	4.74001123	2.70796655
H	-16.33969524	6.79797490	1.75548922
H	-14.75361203	7.34111005	2.20174387
H	-15.99328724	4.62042603	2.68578730
C	-15.39032863	7.46627496	-3.40547748
N	-15.65319028	8.22244858	-2.29446420
C	-15.54953787	7.85024233	-0.96051661
C	-15.03917409	6.50475086	-0.74135622
C	-14.72659376	5.68461605	-1.87520160
N	-14.93176790	6.20866637	-3.14198608
O	-15.86176256	8.63071899	-0.10514819
O	-15.56730109	7.86147414	-4.52085532
H	-16.02827101	9.13112757	-2.46668650
H	-12.47922659	6.33857680	-1.37734730
H	-14.93784565	4.63670760	-1.81108774
H	-14.82663667	5.62795879	-3.94387709
39			
C1_C6_1.5			
C	-10.07222403	-0.00426932	-0.89427794
C	-9.61883900	-0.40999442	-2.14930697
C	-9.83841476	0.43092456	-3.23251519
C	-10.48764603	1.62617681	-3.02761598
C	-10.91284069	1.95397174	-1.73284907
N	-10.70343495	1.14150755	-0.68712994
H	-9.50610195	0.15649079	-4.21683667
H	-9.92204704	-0.62586364	-0.03054106
H	-9.11383513	-1.35026362	-2.26536559
H	-10.66672209	2.29385946	-3.84870489
C	-12.95727723	5.38764912	-1.44387715
C	-12.88134338	4.65858010	-0.13867087
C	-12.13566264	3.52134056	-0.15828792
C	-11.60116876	3.20161235	-1.42829257
S	-11.91278015	4.42590370	-2.59783753
H	-12.01610507	2.85996598	0.67439812
C	-15.21287516	5.09863892	2.82722431
C	-13.80727283	4.64067613	2.36029931
C	-13.76385202	5.11176285	0.92713502
C	-14.82035018	5.88580246	0.58521646
C	-15.72637181	6.10162050	1.76847332
H	-15.18847129	5.52767227	3.82184327
H	-13.68456520	3.56528908	2.46115100
H	-13.01407822	5.10119362	2.94427002
H	-16.76557115	5.94082459	1.50406916
H	-15.64557541	7.13524610	2.08441449
H	-15.87496431	4.24044929	2.86780424
C	-14.93180819	7.43575774	-3.41788797
N	-15.18204799	8.20174399	-2.31802714
C	-15.28723204	7.75671498	-0.99101181

C	-15.02885762	6.38318013	-0.78390231
C	-14.39120689	5.56255996	-1.84792163
N	-14.50297440	6.16786430	-3.15167810
O	-15.61057515	8.56719509	-0.14561582
O	-15.05400126	7.84286680	-4.54090325
H	-15.49780198	9.13018622	-2.49449728
H	-12.50804274	6.36680481	-1.38462354
H	-14.82953381	4.56936779	-1.90311670
H	-14.45851744	5.58756067	-3.95866381
39			
C1_C6_1.6			
C	-10.11853894	-0.03289744	-0.91164263
C	-9.63814651	-0.41848572	-2.16255397
C	-9.81721379	0.44696654	-3.23217283
C	-10.45604508	1.64762390	-3.01886471
C	-10.90915666	1.95313769	-1.72974312
N	-10.73907808	1.11785702	-0.69724368
H	-9.46292265	0.18852170	-4.21314936
H	-10.00001729	-0.67418304	-0.05751892
H	-9.14354740	-1.36345311	-2.28536691
H	-10.60463165	2.33438586	-3.83026942
C	-12.90271182	5.40141158	-1.42565273
C	-12.86413289	4.67318682	-0.13426315
C	-12.14884789	3.51048951	-0.15824140
C	-11.59008276	3.20815008	-1.41521905
S	-11.85741185	4.45751904	-2.56565064
H	-12.06408969	2.83210265	0.66492343
C	-15.20001253	5.10429498	2.83108296
C	-13.78844149	4.66745519	2.36369532
C	-13.75803790	5.12423114	0.92460496
C	-14.82715829	5.88000640	0.58045710
C	-15.73124155	6.09096850	1.76659905
H	-15.18037335	5.53969449	3.82308116
H	-13.64454940	3.59604677	2.47717094
H	-13.00185155	5.15018724	2.93858951
H	-16.76884223	5.91273581	1.50694646
H	-15.66412779	7.12819898	2.07408336
H	-15.84734633	4.23513913	2.87843412
C	-14.94367850	7.42302416	-3.42381247
N	-15.18671583	8.19053986	-2.32596349
C	-15.29200917	7.74858874	-0.99454440
C	-15.05091677	6.37419569	-0.78811627
C	-14.43704283	5.56100189	-1.85034000
N	-14.54842042	6.14396718	-3.15148398
O	-15.59736418	8.57037187	-0.15243931
O	-15.04609002	7.82891630	-4.54942682
H	-15.48401602	9.12525740	-2.50109417
H	-12.51060111	6.40360109	-1.37225184
H	-14.81895587	4.54595197	-1.89046076
H	-14.48864191	5.56187215	-3.95582235
39			

C1_C6_1.7			
C	-10.17018007	-0.07302248	-0.94232832
C	-9.64965182	-0.42916012	-2.18565462
C	-9.77804354	0.46789379	-3.23453656
C	-10.40933315	1.67174881	-3.00958286
C	-10.90341720	1.94581629	-1.72991950
N	-10.78250583	1.08038497	-0.71734474
H	-9.39164119	0.23271481	-4.20922812
H	-10.09137851	-0.73985811	-0.10336125
H	-9.16316660	-1.37711708	-2.31780690
H	-10.52014684	2.38279688	-3.80601747
C	-12.84014916	5.41033089	-1.39852496
C	-12.85620468	4.67275984	-0.13048814
C	-12.17502516	3.48215195	-0.16334759
C	-11.57954675	3.20531866	-1.40172315
S	-11.79017817	4.48845380	-2.52413919
H	-12.13710402	2.78074787	0.64382525
C	-15.18745279	5.12573312	2.83722072
C	-13.78002433	4.68290106	2.36596600
C	-13.76106968	5.12135776	0.92034059
C	-14.82791052	5.88374068	0.57910887
C	-15.71219527	6.11875984	1.77631928
H	-15.16492846	5.55612046	3.83142333
H	-13.63423065	3.61343051	2.49416689
H	-12.98971226	5.17444925	2.92849596
H	-16.75681525	5.96232400	1.53227025
H	-15.61850835	7.15512833	2.07968767
H	-15.83977322	4.25991825	2.87987052
C	-14.96013396	7.40359189	-3.43010780
N	-15.22719733	8.17063496	-2.34088319
C	-15.32982432	7.73675815	-1.00369963
C	-15.06461525	6.36978092	-0.78715197
C	-14.47333152	5.56246565	-1.84525820
N	-14.57907928	6.12072650	-3.14265170
O	-15.64722385	8.56515467	-0.17266046
O	-15.03477285	7.80086606	-4.56075922
H	-15.52622352	9.10386996	-2.52117579
H	-12.50841577	6.43234462	-1.34301227
H	-14.79492290	4.52809662	-1.86852553
H	-14.47697598	5.53844597	-3.94244621
39			
C1_C6_1.8			
C	-10.20273898	-0.11660604	-0.96914572
C	-9.63468495	-0.44280199	-2.20024712
C	-9.71367881	0.48144578	-3.22663591
C	-10.34641977	1.68567818	-2.99431885
C	-10.88879258	1.92802985	-1.72945459
N	-10.81518160	1.03494035	-0.73728537
H	-9.28991826	0.26980898	-4.19115349
H	-10.16279693	-0.80708293	-0.14671596
H	-9.15059389	-1.39120235	-2.33841396

H	-10.41991066	2.41859659	-3.77505781
C	-12.78595343	5.40214899	-1.37891387
C	-12.86833092	4.64974415	-0.13960057
C	-12.20976355	3.43701148	-0.17881453
C	-11.57187433	3.18639527	-1.39329865
S	-11.73241550	4.49803409	-2.49220546
H	-12.21657144	2.71605571	0.61183850
C	-15.17942802	5.15229774	2.83466178
C	-13.79106666	4.66774544	2.35280109
C	-13.78030844	5.09266298	0.90206102
C	-14.82185310	5.90240058	0.57720465
C	-15.65923059	6.19002472	1.79720289
H	-15.14455520	5.55448781	3.84039837
H	-13.67043758	3.59659215	2.49241782
H	-12.98271804	5.14623656	2.90156172
H	-16.71838769	6.11552946	1.58129994
H	-15.48007923	7.21310941	2.10845309
H	-15.86538833	4.31160507	2.85001778
C	-14.99410275	7.39608375	-3.43204294
N	-15.29835772	8.15890466	-2.35347050
C	-15.38724933	7.73674282	-1.00887171
C	-15.07238367	6.38338989	-0.77883380
C	-14.51740184	5.57446159	-1.83977701
N	-14.61655487	6.11091314	-3.12981404
O	-15.72727425	8.56842608	-0.19107090
O	-15.04164479	7.78290597	-4.56706799
H	-15.60210062	9.08946075	-2.53964559
H	-12.50576870	6.43768607	-1.31391212
H	-14.78975771	4.52785466	-1.84345362
H	-14.47732530	5.52884258	-3.92434656
39			
C1_C6_1.9			
	-10.21199270	-0.15999833	-0.97224519
	-9.57842466	-0.46195110	-2.18178510
	-9.60336531	0.47407508	-3.18631380
	-10.25453273	1.68317101	-2.96338624
	-10.86044491	1.89861759	-1.72757851
	-10.83546298	0.98223764	-0.74846777
	-9.12930399	0.28272512	-4.13147835
	-10.21227951	-0.86887571	-0.16432965
	-9.08712803	-1.40827295	-2.31029579
	-10.28663633	2.43024334	-3.73321002
	-12.76133161	5.36868364	-1.39538484
	-12.90330617	4.60311127	-0.17706218
	-12.24265430	3.38463779	-0.20918483
	-11.56461843	3.14978099	-1.40049564
	-11.70183039	4.47075716	-2.49733085
	-12.27522463	2.65601523	0.57378742
	-15.16628981	5.17772474	2.81731798
	-13.82621510	4.60283822	2.30433620
	-13.81585857	5.03669106	0.85486733

	-14.80601800	5.93523403	0.56762621
	-15.56243772	6.28847413	1.82292302
	-15.09601137	5.53627037	3.83771883
	-13.77657986	3.52523596	2.43678715
	-12.97784872	5.02223923	2.84181201
	-16.62951570	6.34865376	1.65063772
	-15.24823343	7.27392335	2.15063984
	-15.91919473	4.39613470	2.80273914
	-15.07298422	7.42444200	-3.42361805
	-15.39424455	8.17930700	-2.34612956
	-15.45050350	7.76519458	-0.99684294
	-15.07677842	6.42488598	-0.76363558
	-14.59187214	5.60465965	-1.84642695
	-14.70539389	6.13133283	-3.12280883
	-15.80187296	8.59144929	-0.18123897
	-15.10952973	7.81063979	-4.55783646
	-15.69828387	9.11032028	-2.53018799
	-12.50232528	6.40853863	-1.31246330
	-14.84483156	4.55564037	-1.82666271
	-14.55167742	5.55378561	-3.91844587
39<br>C1_C6_2.0			
	-10.01721367	-0.14089133	-0.92341642
	-9.50492299	-0.48130576	-2.17118727
	-9.69522413	0.40809409	-3.22056586
	-10.37265568	1.58381904	-2.98529101
	-10.84850244	1.84614120	-1.69721432
	-10.67160166	0.99085424	-0.68872313
	-9.31987765	0.18567507	-4.20308820
	-9.89419742	-0.79863272	-0.08183673
	-8.97896208	-1.40764588	-2.30947028
	-10.53613440	2.28385916	-3.78282886
	-12.67609492	5.36587212	-1.35267918
	-12.87896772	4.59150959	-0.15214746
	-12.23412714	3.32008505	-0.20756554
	-11.56509099	3.09454759	-1.35932882
	-11.63069150	4.45506865	-2.45391609
	-12.29821738	2.58313641	0.56734522
	-15.22211801	5.16988127	2.77409901
	-13.88612061	4.57230945	2.28499107
	-13.82291251	5.01453547	0.83815485
	-14.79004373	5.96669574	0.55422850
	-15.52854286	6.33994773	1.81733967
	-15.18447975	5.47742904	3.81258502
	-13.85827372	3.49309108	2.40752259
	-13.04301055	4.97239709	2.84472671
	-16.58790702	6.48623032	1.64850567
	-15.14087237	7.28328291	2.19073454
	-16.00340853	4.42148071	2.68622649
	-15.09855036	7.48562018	-3.42102931
	-15.47784715	8.21333468	-2.33307158

	-15.49784476	7.80153688	-0.99573643
	-15.04338045	6.46038477	-0.76554115
	-14.58615649	5.65800019	-1.86878152
	-14.67479898	6.21058242	-3.13445761
	-15.87429669	8.58538598	-0.16071907
	-15.14349603	7.89853507	-4.54284486
	-15.79106028	9.14344625	-2.51298814
	-12.42515272	6.40676113	-1.27186376
	-14.81706352	4.60614399	-1.85882769
	-14.51321841	5.64141366	-3.93498002
39			
C1_C6_2.1			
C	-10.38755849	-0.36423504	-1.10441343
C	-9.46747492	-0.52350431	-2.13284768
C	-9.23368791	0.55661246	-2.97435168
C	-9.92163419	1.72967155	-2.76320836
C	-10.82346280	1.80023141	-1.69761010
N	-11.04829839	0.76500302	-0.88799753
H	-8.52420866	0.48018040	-3.77691763
H	-10.60580658	-1.17257135	-0.43063302
H	-8.95648386	-1.45894277	-2.26168667
H	-9.75644118	2.57611262	-3.40168840
C	-12.70459780	5.29186639	-1.45219852
C	-12.94128503	4.52737080	-0.24726827
C	-12.27686842	3.26205730	-0.27061931
C	-11.57087197	3.03485089	-1.39173799
S	-11.64329221	4.36945991	-2.53088155
H	-12.35707283	2.53361850	0.51664462
C	-15.11118104	5.24827049	2.74899791
C	-13.89451799	4.48297295	2.18808078
C	-13.83999872	4.97388839	0.75284739
C	-14.72130795	6.01472696	0.54247424
C	-15.31246641	6.48001454	1.84893130
H	-14.97593476	5.51230587	3.79050998
H	-14.00640534	3.40552076	2.26494531
H	-12.97392194	4.74001123	2.70796655
H	-16.33969524	6.79797490	1.75548922
H	-14.75361203	7.34111005	2.20174387
H	-15.99328724	4.62042603	2.68578730
C	-15.39032863	7.46627496	-3.40547748
N	-15.65319028	8.22244858	-2.29446420
C	-15.54953787	7.85024233	-0.96051661
C	-15.03917409	6.50475086	-0.74135622
C	-14.72659376	5.68461605	-1.87520160
N	-14.93176790	6.20866637	-3.14198608
O	-15.86176256	8.63071899	-0.10514819
O	-15.56730109	7.86147414	-4.52085532
H	-16.02827101	9.13112757	-2.46668650
H	-12.47922659	6.33857680	-1.37734730
H	-14.93784565	4.63670760	-1.81108774
H	-14.82663667	5.62795879	-3.94387709

39			
C1_C6_2.2			
C	-9.99746600	-0.20257100	-0.79440900
C	-9.36298000	-0.54159300	-1.98502100
C	-9.46056200	0.34085500	-3.05186200
C	-10.17174500	1.50889500	-2.88932000
C	-10.77476200	1.76921000	-1.65606100
N	-10.68619800	0.92038200	-0.63100400
H	-8.98896500	0.11963700	-3.99205400
H	-9.94949200	-0.85549500	0.05831700
H	-8.81604800	-1.46210900	-2.06701300
H	-10.26297000	2.20468800	-3.70141900
C	-12.68350900	5.26363400	-1.51770700
C	-12.97386100	4.50335300	-0.31749000
C	-12.28049000	3.24361600	-0.30000000
C	-11.54496500	3.00746100	-1.40177000
S	-11.59763600	4.31831600	-2.56145500
H	-12.35941500	2.53214800	0.49587600
C	-15.16922400	5.15048600	2.67854000
C	-13.97148400	4.38577900	2.08192000
C	-13.88687500	4.92328100	0.66585200
C	-14.73841000	6.01788600	0.49444800
C	-15.30648800	6.42777300	1.82960500
H	-15.04518400	5.36431900	3.73344500
H	-14.11994600	3.31100200	2.12522300
H	-13.05550600	4.59743400	2.63042000
H	-16.31828600	6.79809000	1.75596400
H	-14.70441200	7.24245100	2.22301300
H	-16.06927000	4.55294200	2.57331800
C	-15.36203100	7.67604200	-3.36182200
N	-15.62384300	8.37759400	-2.22209400
C	-15.51327300	7.93562100	-0.90047500
C	-15.04541500	6.58264900	-0.75386900
C	-14.78164000	5.78266300	-1.92829300
N	-14.96564100	6.37416100	-3.15486900
O	-15.78790400	8.70159900	-0.01395700
O	-15.49109300	8.12721200	-4.46047800
H	-15.91004800	9.32467600	-2.34695500
H	-12.41303500	6.29917300	-1.41454000
H	-15.03401200	4.73896400	-1.90132500
H	-14.90891600	5.82635700	-3.98432900
39			
C1_C6_2.3			
C	-10.12894434	-0.29724766	-0.83197416
C	-9.33483031	-0.56276492	-1.94322641
C	-9.26856372	0.39677616	-2.94329550
C	-9.98556804	1.56352781	-2.79689021
C	-10.75864498	1.74529174	-1.64754825
N	-10.82368196	0.82426566	-0.68491502
H	-8.66716467	0.23654530	-3.81969750
H	-10.20975180	-1.01162273	-0.03260535

H	-8.79196263	-1.48652110	-2.01598585
H	-9.94687673	2.32117958	-3.55592664
C	-12.70835061	5.22410304	-1.58915527
C	-13.01775976	4.47121804	-0.37814833
C	-12.29765918	3.22065651	-0.33183836
C	-11.54925171	2.97770408	-1.42117954
S	-11.62007500	4.25783424	-2.61740998
H	-12.37377628	2.52149004	0.47521225
C	-15.11518060	5.18230280	2.66570453
C	-14.00653081	4.33090472	2.01455072
C	-13.92492447	4.88087260	0.60067988
C	-14.73424619	6.03134137	0.46915777
C	-15.18785087	6.47583445	1.83425002
H	-14.92998476	5.37712110	3.71521321
H	-14.24632218	3.27241353	2.05050094
H	-13.06015812	4.45511335	2.53720264
H	-16.16164176	6.94119438	1.82183904
H	-14.48559639	7.22476915	2.19218183
H	-16.06477483	4.66092602	2.59702705
C	-15.47756165	7.69982814	-3.34422386
N	-15.63624805	8.42099017	-2.19881488
C	-15.51320029	7.97180855	-0.87899332
C	-15.09198643	6.60213007	-0.74927235
C	-14.91020367	5.78974385	-1.93828791
N	-15.17270989	6.36866791	-3.14569313
O	-15.72641824	8.75147471	0.01211696
O	-15.62215284	8.15119508	-4.43966190
H	-15.89902966	9.37596254	-2.31583458
H	-12.40577953	6.24953908	-1.46649066
H	-15.15028609	4.74533218	-1.88196636
H	-15.12736095	5.82667848	-3.98026150
39			
C1_C6_2.4			
C	-10.13596753	-0.32833612	-0.80157357
C	-9.29775922	-0.58498360	-1.88198638
C	-9.19004091	0.38392164	-2.86949794
C	-9.91126906	1.55011924	-2.74138267
C	-10.73069587	1.72255670	-1.62298687
N	-10.83521933	0.79262076	-0.67231644
H	-8.55348569	0.23120429	-3.72213256
H	-10.24972435	-1.04992120	-0.01268060
H	-8.75333232	-1.50881537	-1.94123042
H	-9.84002615	2.31545100	-3.49026366
C	-12.66759676	5.20993446	-1.61797501
C	-13.02067469	4.45470019	-0.41292629
C	-12.29679289	3.20256679	-0.34789129
C	-11.52789701	2.95441140	-1.41872738
S	-11.57560482	4.22938833	-2.62846620
H	-12.38875720	2.50664648	0.46044729
C	-15.11239351	5.17890413	2.62328112
C	-14.04518319	4.29090691	1.95332365

C	-13.93979273	4.85930255	0.54711708
C	-14.72273677	6.04380859	0.43694934
C	-15.12437388	6.49057854	1.81825485
H	-14.91773435	5.34464785	3.67600868
H	-14.33226033	3.24387460	1.97133158
H	-13.09383914	4.36376111	2.47615584
H	-16.06800272	7.01284925	1.83474871
H	-14.36959657	7.18621602	2.17738943
H	-16.08597516	4.70500045	2.54598446
C	-15.57285602	7.75270366	-3.33224743
N	-15.70388502	8.45924778	-2.17253484
C	-15.53912668	7.99875054	-0.86334757
C	-15.10177522	6.62594110	-0.76001561
C	-14.96000880	5.82776293	-1.96888102
N	-15.25703578	6.42127156	-3.15980347
O	-15.72857269	8.76630869	0.04172891
O	-15.74947637	8.21975429	-4.41581988
H	-15.97174149	9.41492785	-2.27191195
H	-12.35227211	6.22946567	-1.48085928
H	-15.18209866	4.77983239	-1.91607217
H	-15.22743900	5.89126853	-4.00283044
39			
C1_C6_2.5			
C	-10.13273082	-0.34856989	-0.77780447
C	-9.26415409	-0.60073307	-1.83493171
C	-9.12722697	0.37343504	-2.81376396
C	-9.85088219	1.53955975	-2.70029435
C	-10.70216189	1.70731668	-1.60512590
N	-10.83442185	0.77248212	-0.66265079
H	-8.46641890	0.22467474	-3.64849391
H	-10.26981186	-1.07415624	0.00374333
H	-8.71915859	-1.52487832	-1.88337541
H	-9.75718422	2.30888453	-3.44258310
C	-12.62402262	5.20140449	-1.63993457
C	-13.01794860	4.44400508	-0.44444921
C	-12.29499397	3.18809534	-0.36660104
C	-11.50347708	2.93902119	-1.41816191
S	-11.52123063	4.21627950	-2.63124396
H	-12.40581747	2.49164491	0.43896956
C	-15.10437123	5.17258839	2.58738530
C	-14.07658941	4.25422087	1.89746949
C	-13.94927514	4.84205063	0.50009368
C	-14.71030688	6.05352514	0.41142198
C	-15.06372536	6.49818155	1.80688129
H	-14.90010740	5.31132950	3.64214716
H	-14.40702067	3.21990444	1.89715598
H	-13.12262658	4.27815015	2.41958488
H	-15.97787837	7.06794431	1.85153397
H	-14.26504360	7.14398279	2.16463651
H	-16.09749193	4.74194155	2.50512660
C	-15.67049666	7.79459557	-3.31456235

N	-15.76975632	8.48965613	-2.14336427
C	-15.56037855	8.02123055	-0.84500497
C	-15.11285343	6.64519895	-0.76606534
C	-15.01024198	5.85962221	-1.99022971
N	-15.34458051	6.46347492	-3.16525189
O	-15.72181240	8.78017503	0.07132996
O	-15.88054179	8.27380838	-4.38643366
H	-16.04166049	9.44582989	-2.22665973
H	-12.31506677	6.22163334	-1.49846278
H	-15.20878075	4.80737217	-1.94059749
H	-15.33075202	5.94368770	-4.01510325
39			
C1_C6_2.6			
C	-10.13917330	-0.37032824	-0.76286795
C	-9.24465111	-0.61989479	-1.79862191
C	-9.07585664	0.36137881	-2.76551103
C	-9.79459801	1.53128495	-2.66136601
C	-10.67340631	1.69617142	-1.58761409
N	-10.83653031	0.75441157	-0.65693768
H	-8.39446213	0.21493460	-3.58396081
H	-10.30163260	-1.10127815	0.00879023
H	-8.70455567	-1.54724272	-1.84036753
H	-9.67657309	2.30569664	-3.39486123
C	-12.56879349	5.20087616	-1.64895690
C	-13.00696659	4.44018943	-0.46975058
C	-12.29320211	3.17637881	-0.38481488
C	-11.47171387	2.93132966	-1.41223645
S	-11.44082921	4.22297676	-2.61316845
H	-12.43378184	2.47260960	0.40970722
C	-15.09672179	5.16885595	2.55466085
C	-14.10493397	4.22453877	1.84683273
C	-13.95287333	4.83170130	0.45940246
C	-14.69435853	6.06480330	0.38933584
C	-15.00976902	6.50452763	1.79541731
H	-14.88418570	5.28369434	3.61062228
H	-14.47577292	3.20412168	1.82800938
H	-13.15165557	4.20171488	2.36977545
H	-15.89739792	7.11205037	1.86250121
H	-14.17731927	7.10677413	2.15245198
H	-16.10556903	4.77699813	2.46966933
C	-15.76505774	7.82382861	-3.29772885
N	-15.83134394	8.51217028	-2.11913919
C	-15.57342238	8.04190222	-0.83137202
C	-15.11705193	6.66437117	-0.77227245
C	-15.05100656	5.88715991	-2.00621444
N	-15.42453215	6.49516026	-3.16686132
O	-15.70479535	8.79680444	0.09221725
O	-16.01199610	8.30942319	-4.35863170
H	-16.10848148	9.46791443	-2.18977064
H	-12.28777057	6.22873332	-1.51177636
H	-15.22156533	4.83035158	-1.95772097

H	-15.42867315	5.98144560	-4.02053011
39			
C1_C6_2.7			
C	-10.15395906	-0.39456351	-0.75612347
C	-9.23365796	-0.64144400	-1.76957676
C	-9.02890965	0.34918052	-2.72004895
C	-9.73881523	1.52486484	-2.62211823
C	-10.64547687	1.68647583	-1.57120513
N	-10.84323547	0.73581442	-0.65648911
H	-8.32699322	0.20521080	-3.52137977
H	-10.34470852	-1.13245984	0.00239845
H	-8.70173172	-1.57373357	-1.80733130
H	-9.59334915	2.30585136	-3.34366715
C	-12.51089412	5.20145314	-1.65145695
C	-12.99391891	4.43818428	-0.49367705
C	-12.29495876	3.16396479	-0.40596390
C	-11.43779578	2.92651168	-1.40435124
S	-11.34674124	4.23973503	-2.58014978
H	-12.47220942	2.44886438	0.37081130
C	-15.08664009	5.16789972	2.52418051
C	-14.13109734	4.19922349	1.79866631
C	-13.95429782	4.82468010	0.42166270
C	-14.67454787	6.07704915	0.36978643
C	-14.95487951	6.51116183	1.78461436
H	-14.86627026	5.25975693	3.58070757
H	-14.54158851	3.19441313	1.76248153
H	-13.18030461	4.13017196	2.32171954
H	-15.81546094	7.15346240	1.87240204
H	-14.09214024	7.07072150	2.13970073
H	-16.10938917	4.81418427	2.43792245
C	-15.85976357	7.84622389	-3.27831960
N	-15.88308334	8.53319303	-2.09661346
C	-15.57481557	8.06413570	-0.82010970
C	-15.11583926	6.68488975	-0.77813085
C	-15.08993435	5.91194804	-2.01717343
N	-15.50662846	6.51993065	-3.16292751
O	-15.67106837	8.81867078	0.10753950
O	-16.14904885	8.33321125	-4.32775348
H	-16.16563768	9.48810835	-2.15623250
H	-12.27721131	6.24185773	-1.53166533
H	-15.23030218	4.85100988	-1.96814061
H	-15.53570559	6.00874631	-4.01764813
39			
C1_C6_2.7			
C	-10.17557198	-0.42224507	-0.75926172
C	-9.22197416	-0.66158565	-1.74312743
C	-8.97443273	0.34249385	-2.66915798
C	-9.67659416	1.52313816	-2.57689437
C	-10.61839782	1.67680848	-1.55595189
N	-10.85736216	0.71323167	-0.66500598
H	-8.24564425	0.20480489	-3.44733722

H	-10.40019810	-1.17051914	-0.02045303
H	-8.69740557	-1.59809660	-1.77736297
H	-9.49893223	2.31384563	-3.28047848
C	-12.45376374	5.19993258	-1.65045157
C	-12.98095900	4.43574766	-0.51834931
C	-12.30002854	3.14990541	-0.43075814
C	-11.40433363	2.92154928	-1.39606240
S	-11.24834761	4.25948647	-2.53682529
H	-12.51724391	2.42194548	0.32366287
C	-15.07389420	5.16926177	2.49508446
C	-14.15607744	4.17739250	1.75108147
C	-13.95513318	4.81894045	0.38441163
C	-14.65162167	6.08884486	0.35001362
C	-14.90200272	6.51697057	1.77157668
H	-14.84300326	5.24072806	3.55093288
H	-14.60555448	3.19000177	1.69926479
H	-13.20882743	4.06263612	2.27225098
H	-15.73797409	7.18840878	1.87672948
H	-14.01517434	7.03911558	2.12415676
H	-16.10876420	4.85192250	2.41260086
C	-15.96169093	7.86228458	-3.25476462
N	-15.93074284	8.55275092	-2.07466167
C	-15.56338972	8.08828719	-0.81233084
C	-15.10797644	6.70685126	-0.78611982
C	-15.12963104	5.93422079	-2.02535822
N	-15.59688945	6.53832950	-3.15337501
O	-15.61499603	8.84624224	0.11608356
O	-16.30082440	8.34669476	-4.29043651
H	-16.21583993	9.50753177	-2.12421453
H	-12.28196409	6.25446294	-1.56342992
H	-15.23979817	4.86997660	-1.97361240
H	-15.66002037	6.02624139	-4.00570874
